# The PANoptosis-related signature indicates the prognosis and tumor immune infiltration features of gliomas

**DOI:** 10.3389/fnmol.2023.1198713

**Published:** 2023-07-12

**Authors:** Jingjing Song, Zekun Xu, Qingchen Fan, Yanfei Sun, Xiaoying Lin

**Affiliations:** ^1^The Second Hospital, Cheeloo College of Medicine, Shandong University, Jinan, Shandong, China; ^2^Medical Integration and Practice Center, Cheeloo College of Medicine, Shandong University, Jinan, Shandong, China; ^3^Department of Neurosurgery, Qilu Hospital, Cheeloo College of Medicine and Institute of Brain and Brain-Inspired Science, Shandong University, Jinan, China; ^4^Jinan Microecological Biomedicine Shandong Laboratory and Shandong Key Laboratory of Brain Function Remodeling, Jinan, China

**Keywords:** glioma, PANoptosis, PANoptosome, prognosis, immune cell infiltration

## Abstract

**Background:**

Gliomas are the most common primary tumors of the central nervous system, with high heterogeneity and highly variable survival rates. Accurate classification and prognostic assessment are key to the selection of treatment strategies. One hallmark of the tumor is resistance to cell death. PANoptosis, a novel mode of programmed cell death, has been frequently reported to be involved in the innate immunity associated with pathogen infection and played an important role in cancers. However, the intrinsic association of PANoptosis with glioma requires deeper investigation.

**Methods:**

The genetics and expression of the 17 reported PANoptosome-related genes were analyzed in glioma. Based on these genes, patients were divided into two subtypes by consensus clustering analysis. After obtaining the differentially expressed genes between clusters, a prognostic model called PANopotic score was constructed after univariate Cox regression, LASSO regression, and multivariate Cox regression. The expression of the 5 genes included in the PANopotic score was also examined by qPCR in our cohort. The prognostic differences, clinical features, TME infiltration status, and immune characteristics between PANoptotic clusters and score groups were compared, some of which even extended to pan-cancer levels.

**Results:**

Gene mutations, CNVs and altered gene expression of PANoptosome-related genes exist in gliomas. Two PANoptotic clusters were significantly different in prognosis, clinical features, immune characteristics, and mutation landscapes. The 5 genes included in the PANopotic score had significantly altered expression in glioma samples in our cohort. The high PANoptotic score group was inclined to show an unfavorable prognosis, lower tumor purity, worse molecular genetic signature, and distinct immune characteristics related to immunotherapy. The PANoptotic score was considered as an independent prognostic factor for glioma and showed superior prognostic assessment efficacy over several reported models. PANopotic score was included in the nomogram constructed for the potential clinical prognostic application. The associations of PANoptotic score with prognostic assessment and tumor immune characteristics were also reflected at the pan-cancer level.

**Conclusion:**

Molecular subtypes of glioma based on PANoptosome-related genes were proposed and PANoptotic score was constructed with different clinical characteristics of anti-tumor immunity. The potential intrinsic association between PANoptosis and glioma subtypes, prognosis, and immunotherapy was revealed.

## Graphical Abstract

Image created using Figdraw.

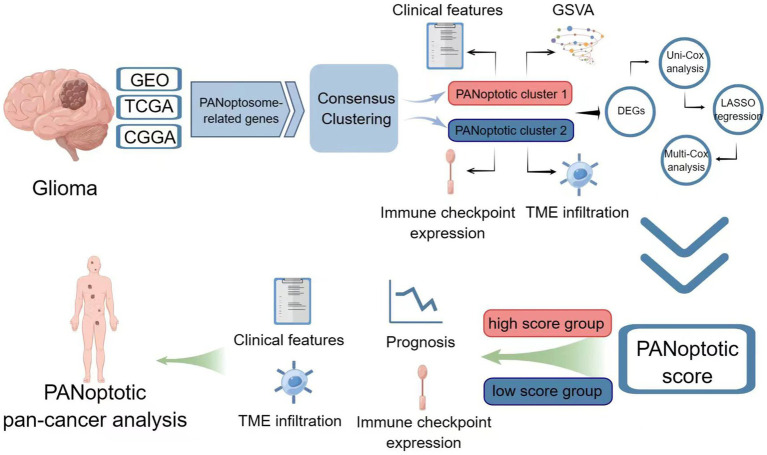

## 1. Introduction

Glioma is one of the primary brain tumors originating from glial cells or precursor cells (Weller et al., 2015; Lapointe et al., 2018). Glioma is the most common type of central nervous system (CNS) primary tumor (Perry and Wesseling, 2016; Reifenberger et al., 2017), accounting for about 75% of malignant primary brain tumors in adults (Lapointe et al., 2018). In general, the incidence of glioma increases with age (Weller et al., 2015), with an annual incidence of about 3–8 per 100,000 people (Xu et al., 2022). Because glioma is highly heterogeneous, prognosis and survival rates vary widely according to the grade of malignancy and molecular characteristics, with 5-year survival rates ranging from 94% in WHO I to 5.5% in WHO IV (Lapointe et al., 2018). With the intratumoral heterogeneity of glioma (Reifenberger et al., 2017; Nicholson and Fine, 2021), the high plasticity of malignant tumor cells (Nicholson and Fine, 2021; Varn et al., 2022), the relatively immunosuppressive environment protected by the blood–brain barrier (Lapointe et al., 2018; McKinnon et al., 2021), and many other factors, glioma is easily resistant to existing treatment techniques such as surgery and radiotherapy (Reifenberger et al., 2017; Varn et al., 2022). It has been shown that most diffuse low-grade gliomas and almost all high-grade gliomas will recur with progression to higher-grade gliomas (Lapointe et al., 2018). The prognosis for glioma with the available treatments is not ideal.

Accurate classification of glioma is crucial to the reliability of prognosis judgment and the best choice of treatment (Horbinski et al., 2022). In 2007, the WHO’s understanding of glioma was still limited to the histopathological level. This classification was limited by the variability of interobserver, and there was inconsistent biological behavior within the same classification, leading to significant differences in clinical outcomes (Nicholson and Fine, 2021; Horbinski et al., 2022). As understanding of the molecular basis of glioma has improved, in 2016 WHO combined traditional histopathology with diagnostic genetics (Nicholson and Fine, 2021), and for the first time incorporated the molecular features of *IDH* mutation and 1p/19q co-deletion into the diagnosis of glioma (Wesseling and Capper, 2018; Horbinski et al., 2022). To a certain extent, it reduced the bias caused by observer subjectivity and provided guidance for the prognosis and targeted treatment of glioma. The fifth edition of the Central Nervous System Tumor Classification released in 2021 expands the classification system introduced in the 2016 edition and makes the comprehensive diagnosis of gliomas based on histopathological characteristics, WHO classification, and molecular information now (Gusyatiner and Hegi, 2018; Horbinski et al., 2022). Although this new classification marks the rapid development of research on the molecular phenotype of glioma, it still has challenges in clinical implementation due to the differences in molecular diagnosis levels in different regions (Horbinski et al., 2022). Therefore, we urgently need to link histopathology, genetics, epigenetics, and transcriptome with clinical information (Nicholson and Fine, 2021), redefine the molecular subtype of glioma and provide more feasible opportunities and options for the prognosis and treatment of glioma.

One hallmark of tumors is their resistance to cell death (Malireddi et al., 2021; Mall et al., 2022). Cells have multiple death pathways to maintain physiological homeostasis, whether the cells are in a normal or stressed state, programmed cell death (PCD, includes apoptosis, pyroptosis, necroptosis, ferroptosis, autophagy, and PANoptosis) and non-PCD (Moujalled et al., 2021; Liu et al. J. 2022). The inactivation of PCD contributes to the progression of brain tumors and affects the therapeutic effect (Moujalled et al., 2021). PANoptosis is one of the PCD pathways regulated by the PANoptosome, integrating multiple components of other PCD pathways. PANoptosis is a unique innate immune-mediated inflammatory pathway (Samir et al., 2020; Malireddi et al., 2021; Gullett et al., 2022; Liu et al. J. 2022; Mall et al., 2022; Pandian and Kanneganti, 2022). PANoptosis has been shown in numerous studies to play a role when the body is infected by pathogens such as bacteria and viruses, inducing inflammatory cell death (Lee et al., 2021). A growing body of evidence confirms that PANoptosis is equally important in tumors (Mall et al., 2022). For example, IRF1 activates PANoptosis to prevent the occurrence of colorectal cancer (Karki et al., 2020; Gullett et al., 2022; Mall et al., 2022); the combination of IFN and a nuclear export inhibitor can reduce the volume of melanoma by upregulating PANoptosis (Gullett et al., 2022; Mall et al., 2022; Pandian and Kanneganti, 2022); TNF-α and IFN-γ combine to induce PANoptosis, leading to the death of 13 types of tumor cells, including colon, lung, and melanoma (Malireddi et al., 2021; Lin et al., 2022; Mall et al., 2022; Pandian and Kanneganti, 2022). If one or more PCD pathways are blocked, PANoptosis activates the alternative cell death defense mechanism, playing an important role in the escape of tumor cells from PCD and anti-tumor therapy (Samir et al., 2020; Pan et al., 2022). The function and mechanism of PANoptosis in glioma still need further investigation.

In this study, we proposed the molecular subtypes of glioma based on PANoptosome-related genes and constructed the PANoptotic score with different clinical characteristics of anti-tumor immunity. Moreover, we revealed the altered genetics and expression of PANoptosome-related genes in glioma samples, explored the relationships between PANoptotic subtypes or models and glioma prognosis, tumor purity, molecular genetic characteristics, immune checkpoint expression, and other immune features related to immunotherapy, and revealed the potential intrinsic link between PANoptosis and heterogeneity, prognosis and immunotherapy of glioma.

## 2. Materials and methods

### 2.1. The source and preprocessing of glioma datasets

The first cohort we used was derived from Gene Expression Omnibus (GEO, https://www.ncbi.nlm.nih.gov/geo/), including four datasets of gliomas, GSE4290 (*n* = 180, 23 normal and 157 tumors), GSE16011 (*n* = 284, 8 normal and 276 tumors), GSE43378 (*n* = 50) and GSE43289 (*n* = 40). Clinical features and survival status as well as microarray data were obtained. We deleted the repeated cases and selected the tumor cases with complete survival data in GSE4290 (*n* = 157), GSE16011 (*n* = 264), GSE43378 (*n* = 50), and GSE43289 (*n* = 33). The normal and tumor tissue data from GSE4290 and GSE16011 were used for principal component analysis (PCA) and the comparison of expression differences between controlled and glioma groups. The tumor tissue data in GSE16011, GSE43378, and GSE43289 were used for further analysis.

Another cohort was obtained from The Cancer Genome Atlas (TCGA) downloaded from Genomic Data Commons Data Portal (GDC, https://portal.gdc.cancer.gov/) including two glioma datasets, TCGA-LGG and TCGA-GBM. We acquired both the RNA-Seq data (count value) and detailed clinical and survival information. After removing the repeated cases, we selected the primary cases with complete survival information in TCGA-LGG (*n* = 510) and TCGA-GBM (*n* = 153). The count values were transformed into transcripts per million (TPM) values.

We also downloaded the mRNA sequencing data (fragments per kilobase million, FPKM value) and corresponding clinical data of another two glioma datasets, mRNAseq-693 (*n* = 693) and mRNAseq-325 (*n* = 325), from Chinese Glioma Genome Atlas (CGGA, http://www.cgga.org.cn/). We also converted FPKM values to TPM values. When primary glioma patients were included and patients without survival information were removed from further evaluation, there remained 404 and 222 samples in the two datasets, respectively.

The gene intersection of GEO, TCGA, and CGGA expression data was obtained using R software, and a total of 15,097 genes in the intersection were collected for further analysis. The rank transformation was performed on all the expression data from GEO, TCGA, and CGGA (Conover and Iman, 1981).

We also downloaded the single nucleotide variation (SNV) and copy number variation (CNV) data of TCGA-LGG and TCGA-GBM from GDC. We used the waterfall function in the “maftools” R package to present the mutation landscape and Gistic2.0 was applied for CNV analysis. And RNA sequencing data (TPM value) of 33 kinds of tumors in TCGA were used for pan-cancer analysis after rank transformation.

### 2.2. Human clinical specimens

3 pairs of glioma tissues and adjacent non-tumor tissues were collected from the department of neurosurgery, Qilu Hospital of Shandong University. This study was approved by the Ethical Committee of Medical Integration and Practice Center, Shandong University, and informed consent from the patients was obtained.

### 2.3. Defining PANoptosome-related genes

In previous research, Christgen et al. (2020) pointed out that there is a single cell death complex that acts as a molecular platform triggering pyroptosis, apoptosis, and necroptosis (PANoptosis) and named this complex PANoptosome. Initially, they found during the activation of PANoptosis by bacterial and viral triggers, RIPK1, RIPK3, CASP8, NLRP3, ASC, and FADD can interact to form a PANoptosome (Christgen et al., 2020). Later, Place et al. (2021) published a review focused on PANoptosis in microbial infection and mentioned ZBP1 is critical to PANoptosis in response to viral pathogens and caspase-1, caspase-11, RIPK3, and caspase-8 are also key PANoptotic components. Babamale and Chen (2021) illustrated that NLRP3 inflammasome activation plays a vital role in PANoptosis during microbial and parasitic infections and provided a PANoptosome model including NLRP3, ZBP1, ASC, Pro-caspase1, caspase-6, caspase-8, FADD, cFLIP, and RIPK3. Then Sundaram and Kanneganti (2021) pointed out that in addition to NLRP3, the best-characterized inflammasome sensor, NLRP1, NLRC4, NLRP6, NLRP9, AIM2, and Pyrin can also act as sensors to form inflammasomes and specifically emphasized that NLRC4 inflammasome may play a crucial role in PANoptosis. Sharma and Kanneganti (2023) described the role of these inflammasomes in colorectal cancer and mentioned that inflammasomes can also act as indispensable components of PANoptosome. We therefore selected the 17 genes (*AIM2, CASP1, CASP4, CASP6, CASP8, CFLAR, FADD, MEFV, NLRC4, NLRP1, NLRP3, NLRP6, NLRP9, PYCARD, RIPK1, RIPK3*, and *ZBP1*) as PANoptosome-related genes.

### 2.4. Consensus clustering

In GEO, TCGA, and CGGA cohorts, we applied consensus clustering to distinct different PANoptosome-related genes expression patterns using the k-means method, respectively. Consensus clustering was performed by the “ConsensusClusterPlus” R package and 1,000 repetitions were conducted to guarantee the stability of our classification (Hartigan and Wong, 1979; Wilkerson and Hayes, 2010). The optimal cluster number was determined by “Nbclust” R packages (Charrad et al., 2014).

### 2.5. Enrichment analysis

“GSVA” R package was used to conduct the gene set variation analysis (GSVA) (Hänzelmann et al., 2013). We downloaded “c5.go.v7.5.1.symbols,” “c2.cp.kegg.v7.5.1.symbols” and “h.all.v7.5.1.symbols” from the Molecular Signature Database (MSigDB, http://www.gsea-msigdb.org/gsea/msigdb) to perform Gene Ontology (GO), Kyoto Encyclopedia of Genes and Genomes (KEGG) and HALLMARKS enrichment analysis, respectively. The GSVA differences between high and low PANoptototic score groups and different clusters were evaluated by the “limma” R package, and an adjusted *p* < 0.05 was considered to indicate statistical significance. And gene set enrichment analysis (GSEA) was implemented by “clusterProfiler” R package (Subramanian et al., 2005).

### 2.6. Tumor microenvironment cell (TME) infiltration

CIBERSORTx algorithm and EPIC were used to quantify the proportions of immune cell infiltration. For CIBERSORTx, our gene expression data was uploaded to the CIBERSORTx website^1^ and LM22 was used as a signature matrix file with 1,000 permutations (Newman et al., 2015). The results of EPIC were also obtained from the EPIC web portal^2^ (Racle and Gfeller, 2020). The “ESTIMATE” R package was used to estimate the tumor purity scores (Yoshihara et al., 2013).

### 2.7. The establishment and assessment of the risk model

We choose GEO datasets (*n* = 347) as a training cohort and the “limma” R package was applied to obtain the differentially expressed genes (DEGs, adjusted *p* value <0.05 and absolute value of log2 (FC) > log2 (1.5)) between different clusters. Then 450 DEGs were included in the univariate Cox regression performed by the “survival” R package, and those genes with a *p* value <0.05 in univariate Cox regression (*n* = 444) were included in the LASSO regression conducted by the “glmnet” R package. We used lambda.min to obtain the optimal model through LASSO analysis. Then 18 genes were included in the multivariate Cox regression performed by the “survival” R package and a *p* value <0.05 was selected as a statistical boundary. Finally, we obtained a risk score model including 5 genes as follows:


$$\begin{array}{c} \text{PANoptotic score}=\left(0.170008438280127\times MEOX2\;\text{expression level}\right)\\ \;+\left(0.196870811688214\times IBSP\;\text{expression level}\right)\\ \;+\left(-0.431404278334841\times GFRA1\;\text{expression level}\right)\\ \;+\left(-0.34193718212311\times NOG\;\text{expression level}\right)\\ \;+\left(0.292158077431546\times EPHB1\;\text{expression level}\right) \end{array}$$

The median value of the PANoptotic score in the training cohort (0.3658584) was used as the cutoff point to divide the patients into a high PANoptotic score group and a low PANoptotic score group. We used Kaplan–Meier survival curves to assess the performance of this model in distinguishing different subtypes of patients and the time-dependent receiver operator characteristic curve (ROC) to evaluate the efficacy of this model.

### 2.8. Construction and validation of a nomogram

Univariate and multivariate Cox regressions were used to determine the independent prognostic value of the PANoptotic score and other clinical characteristics. Those items with *p* < 0.05 both in the univariate and multivariate were considered as independent prognostic factors and included in nomogram construction. The “survival” and “rms” R package were used to construct a nomogram for predicting overall survival (OS). The prediction efficacy of the nomogram was assessed by C-index, which ranges from 0.5 to 1.0; the closer the C-index is to 1.0, the more precise the prediction efficacy of the nomogram is. Comparing the predicted probabilities with the actual probabilities, we can obtain the Calibration curves for the 1-, 3-, and 5-year OS to evaluate the performance of the nomogram; an ideal Calibration curve falls along the 45-degree line.

### 2.9. RNA extraction and real-time PCR

Total RNA of clinical samples was extracted using the TRIzol method following the manufacturer’s protocol (Invitrogen, United States). Complementary DNA (cDNA) was synthesized using the PrimeScript RT Reagent Kit with gDNA Eraser (Takara, Japan). The expression levels of the 5 genes were verified by real-time PCR using TB Green Premix Ex Taq (Takara, Japan). Primer sequences are listed in Supplementary Table S1.

### 2.10. Statistical analysis

Kaplan–Meier method was used for survival analysis and the intergroup differences in survival curves were compared using the log-rank test. Time-dependent ROC was performed using “timeROC” R package and the “compare” function in the “timeROC” package was applied to compare the area under the curve (AUC) of two time-dependent ROC. All the data analyses were carried out in the R4.2.0, GraphPad Prism9, and SPSS28 software. The Chi-square test was used for the comparison of proportion between the two groups and the Wilcoxon test or Student’s *t*-test was used to compare the mean value between the two groups; all *p* values are two-tailed. Statistical significance was described as follows: ns, not significant; ^*^, *p* < 0.05; ^**^, *p* < 0.01; ^***^, *p* < 0.001; ^****^, *p* < 0.0001.

## 3. Results

### 3.1. An overview of genetic variation and altered expression of PANoptosome-related genes in glioma

**Figure 1:**
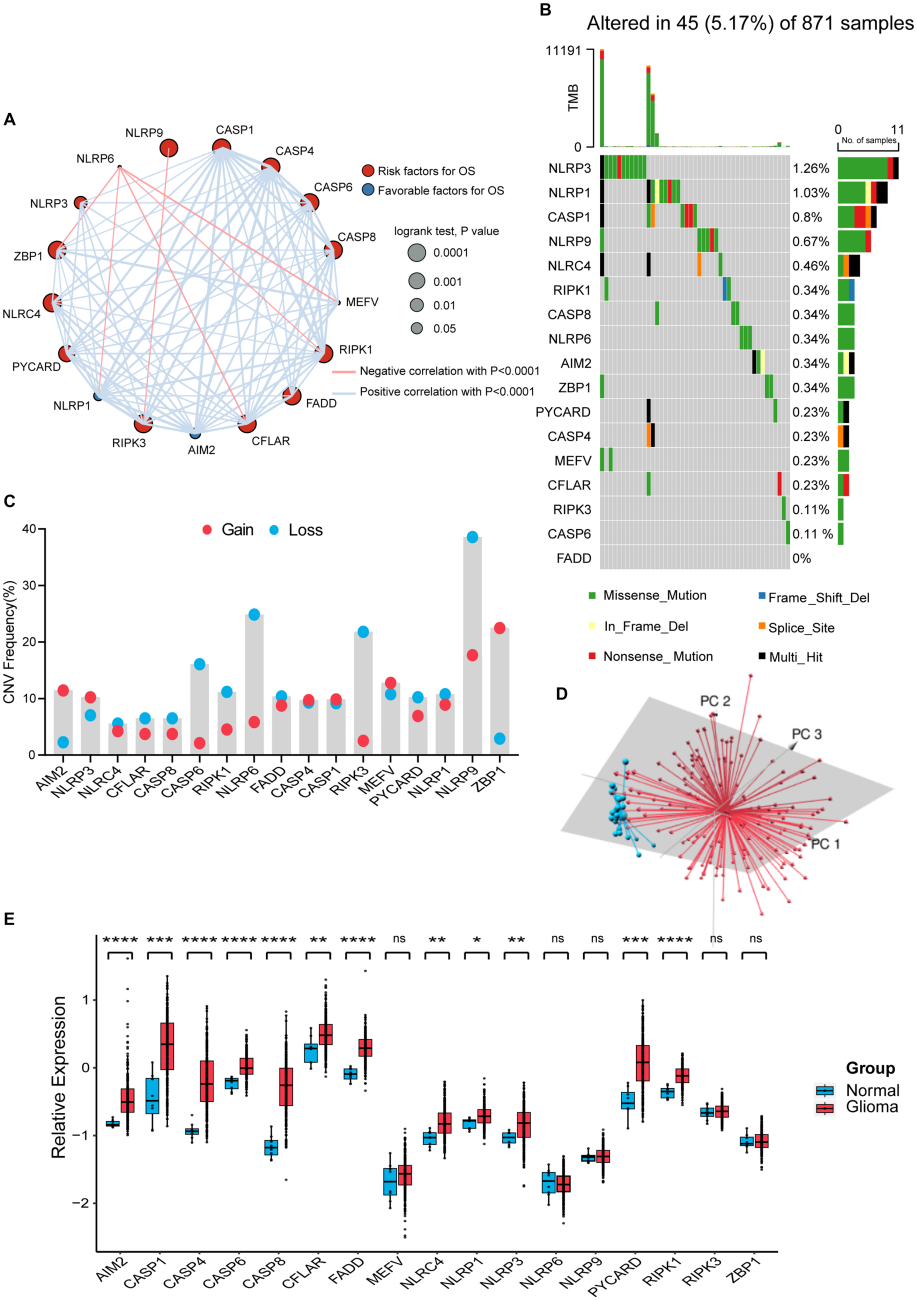
Characterization and alteration of PANoptosome-related genes in glioma. **(A)** An aggregate of the potential biological interaction of PANoptosome-related genes in glioma. The circle size represented the effect of each gene on the prognosis, and the range of *p* values calculated by the Log-rank test was *p* < 0.0001, *p* < 0.001, *p* < 0.01, and *p* < 0.05, respectively. Red dots in the circle are risk factors of prognosis and blue dots in the circle are favorable factors of prognosis. The lines linking genes showed their interactions, and thickness means the correlation strength between them. The positive correlation was marked with red and the negative correlation with blue. **(B)** The mutation status in 871 glioma patients from TCGA-LGG and TCGA-GBM cohorts. Every waterfall plot represented mutation information of each PANoptosome-related gene. Different colors had corresponding annotations at the bottom which mean different mutation types. The above barplot indicated mutation burden. The right numbers presented mutation frequency individually. **(C)** Copy number variations (CNVs) frequency of PANoptosome-related genes in TCGA-LGG and TCGA-GBM cohorts. The proportions of different types were shown by the height of the columns. **(D)** Principal component analysis (PCA) to distinguish gliomas (*n* = 157) from normal samples (*n* = 23) in the GSE4290 cohort. **(E)** The expressions of PANoptosome-related genes between normal tissues (*n* = 8) and glioma tissues (*n* = 276) in the GSE16011 cohort (Wilcoxon test, ^*^p < 0.05; ^**^p < 0.01; ^***^p < 0.001; ^****^p < 0.0001; ns, not significant).

We selected 17 genes mentioned in Materials and Methods as PANoptosome-related genes and investigated their potential biological interaction and prognostic value in glioma. The expression of *NLRP6* was negatively correlated with the expression of *ZBP1*, *NLRP1*, *CFLAR*, *RIPK1* and *MEFV*, and *NLRP9* was also negatively correlated with *RIPK3*, while the correlations between other remaining genes were positive. Most of these genes are risk factors for glioma, but *NLRP1* and *AIM2* have favorable effects on prognosis of glioma (Figure 1A). At the genetic level, we first studied the mutation status of PANoptosome-related genes in glioma patients (Figure 1B). Among the 871 samples, 45 samples showed mutations of PANoptosome-related genes, accounting for 5.17%. *NLRP3* had the highest mutation frequency, and the mutation frequency of *NLRP3*, *NLRP1*, *CASP1*, and *NLRP9* were all greater than 0.5%, and the most common type of mutation was missense mutation, but we did not observe any mutation of *FADD*. In addition, we studied the copy number variation of PANoptosome-related genes in glioma (Figure 1C). Most of the genes mainly showed copy number deletion, while *AIM2* and *ZBP1* mainly showed copy number amplification. Based on the expression of PANoptosome-related genes, we can distinguish tumor samples from normal samples of glioma by principal component analysis (PCA) (Figure 1D and Supplementary Figure S1A). Compared with normal tissues, except for *MEFV*, *NLRP6*, *NLRP9*, *RIPK3*, and *ZBP1*, the expression levels of the remaining genes were relatively high in glioma tissues (Figure 1E and Supplementary Figure S1B). The results above showed the characteristics and differences of PANoptosome-related genes in glioma, which may partly reflect the heterogeneity and intrinsic molecular characteristics of glioma.

### 3.2. Identification of a glioma classification pattern mediated by 17 PANoptosome-related genes

**Figure 2:**
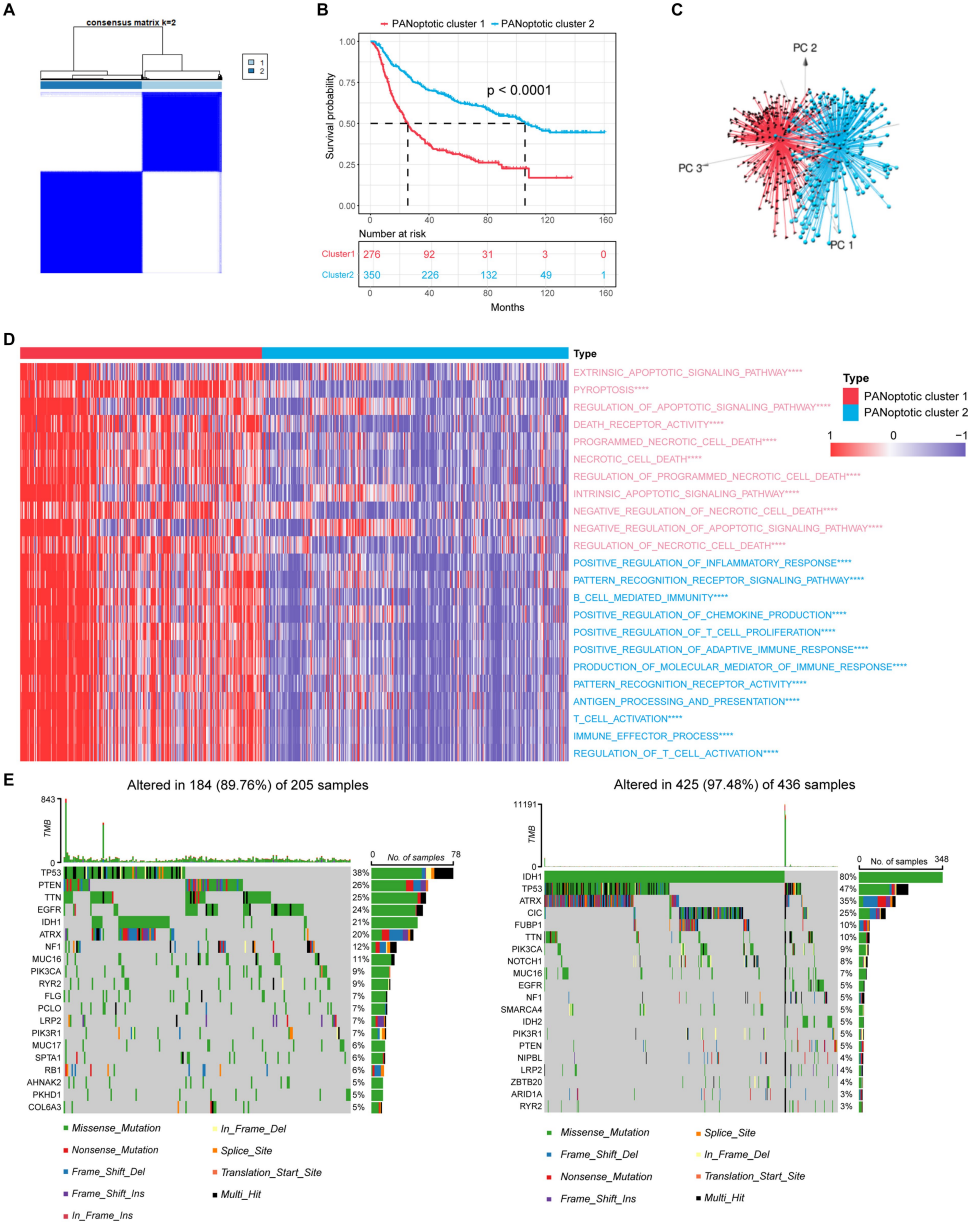
Molecular subtypes of glioma are divided by PANoptosome-related genes. **(A)** The consensus score matrix of patients with glioma in CGGA cohorts (CGGA-693 and CGGA-325) when k = 2. Two samples would be inclined to be grouped into the same cluster if a higher consensus score was observed between them in different iterations. **(B)** OS curves for the two PANoptotic clusters based on 626 patients with glioma from CGGA cohorts (CGGA-693 and CGGA-325) (Log-rank test, *p <* 0.0001). OS, Overall survival. **(C)** Principal component analysis (PCA) to distinguish cluster 1 (*n* = 276) from cluster 2 (*n* = 350) in CGGA cohorts (CGGA-693 and CGGA-325). **(D)** Heatmap differences of GSVA-based GO enrichment analysis between the two PANoptotic clusters in CGGA cohorts (CGGA-693 and CGGA-325) (Student’s *t*-tests, ^****^p < 0.0001). **(E)** The mutation landscape of PANoptotic cluster 1 and PANoptotic cluster 2 in TCGA cohorts (TCGA-LGG and TCGA-GBM).

Based on the expression levels of 17 PANoptosome-related genes, we identified two distinct molecular subtypes by using the unsupervised clustering method, including 276 cases in PANoptotic cluster 1 and 350 cases in PANoptotic cluster 2 (Figure 2A and Supplementary Figures S1D–G). Next, to assess the optimal number of clusters, we used the “NbClust” R package and found that k = 2 was the best cluster number (Supplementary Figure S1C). The survival advantage of PANoptotic cluster 1 was lower than that of PANoptotic cluster 2 (Figure 2B). Subsequently, we confirmed that the gene expression of the two molecular subtypes divided based on the 17 PANoptosome-related genes could be distinguished clearly (Figure 2C). The analogous results of principal component analysis and survival analysis are also received from the TCGA and GEO datasets (Supplementary Figures S1H–K). We included PANoptotic cluster and clinical characteristics, such as age, histology, *IDH* mutation, 1p/19q co-deletion, and MGMT promoter methylation into multivariate Cox regression analysis and found that PANoptotic cluster is an independent factor affecting the prognosis of glioma patients (Supplementary Figure S2A). To explore the differences in biological behavior between these two clusters, we also conducted GSVA enrichment analysis (Figure 2D and Supplementary Figures S2B,C). The results of GO enrichment analysis showed that a variety of signaling pathways related to the immune response, pyroptosis, apoptosis, and necroptosis pathways were enriched in cluster 1 cases. Lastly, we analyzed the distribution differences of somatic mutation between two PANoptotic clusters in the TCGA dataset using the **“**maftools**”** package. PANoptotic cluster 2 presented a more extensive tumor mutation burden than cluster 1, with the rate of the total significant mutated gene at 97.48% versus 89.76% (Figure 2E). Although PANoptotic cluster 2 has more mutations than PANoptotic cluster 1, PANoptotic cluster 2 has a better prognosis. To explain this result, we explored further two clusters of genes with high mutation frequencies. *TP53* is the most frequently mutated gene in human cancers (Boutelle and Attardi, 2021). *PTEN* is also a tumor suppressor gene, and mutations in it lead to dysregulation of the relevant pathways, resulting in overgrowth (Yehia et al., 2020). While the gene with the highest mutation frequency in PANoptotic cluster 2 was *IDH1*. *IDH* mutant gliomas tend to be less aggressive compared to wild-type gliomas of the same WHO classification (Horbinski, 2013). We can therefore tentatively speculate that it may not be the number of mutations but the type of mutation that affects the prognosis.

### 3.3. Differences in clinical characteristics and TME infiltration between two PANoptotic clusters

**Figure 3:**
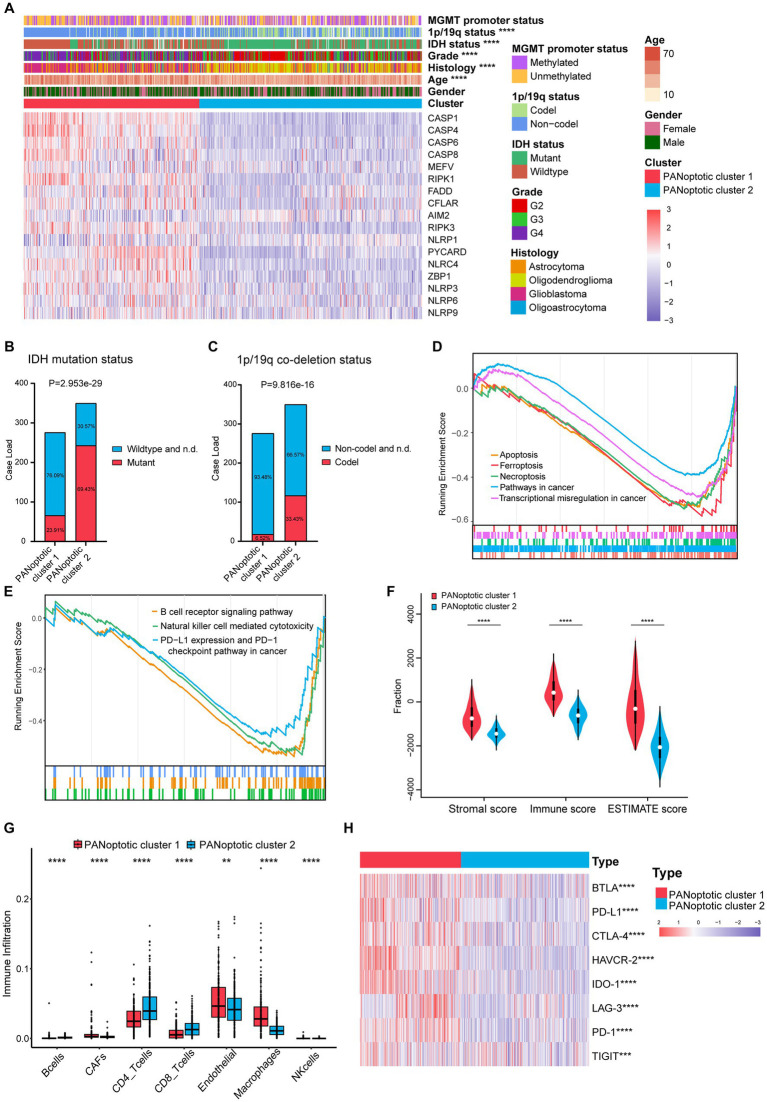
Different PANoptotic clusters showed diverse clinical features and TME infiltration. **(A)** Unsupervised clustering of PANoptosome-related genes in the CGGA cohorts (CGGA-693 and CGGA-325). The PANoptotic cluster, gender, age, histology, WHO grade, *IDH* mutation status, 1p19q co-deletion status, and MGMT promoter methylation status were used as patient annotations. Red indicated high expression of genes and blue indicated low expression (Chi-square test, ^****^p < 0.0001). **(B,C)** The *IDH* mutation status and 1p19q co-deletion status of PANoptotic cluster 1 (*n* = 276) and PANoptotic cluster 2 (*n* = 350) in CGGA cohorts (CGGA-693 and CGGA-325) (Chi-square test). **(D,E)** Gene set enrichment analysis (GSEA) in the combined GEO cohort (GSE16011, GSE43378, and GSE43289). **(F)** Different PANoptotic clusters showed diverse immune scores by ESTIMATE in CGGA cohorts (CGGA-693 and CGGA-325) (Student’s *t*-test, ^****^p < 0.0001). **(G)** The abundance of TME infiltrating cells between the two PANoptotic clusters was analyzed by EPIC in CGGA-693 (Wilcoxon test, ^**^p < 0.01; ^****^p < 0.0001). (H) Unsupervised clustering of immune checkpoint genes between the two PANoptotic clusters in CGGA cohorts (CGGA-693 and CGGA-325) (Student’s t-test, ^***^p < 0.001; ^****^p < 0.0001).

To evaluate the clinical significance of the new glioma classification pattern, we compared the differences in clinical characteristics between the two PANoptotic clusters. The heatmap results showed that most genes were expressed at high levels in PANoptotic cluster 1 while there are still several genes presenting a relatively low expression level in cluster 1, such as *AIM2*. Moreover, we noticed that patients with *IDH* mutant, 1p/19q co-deletion, and low-grade glioma were associated with PANoptotic cluster 2 and that patients with *IDH* wide type, 1p/19q non-codeletion, and glioblastoma were mainly associated with the PANoptotic cluster 1, which contributes to the PANoptotic cluster 2 was linked to a better survival advantage (Figures 3A–C and Supplementary Figures S3A–D). To determine whether the PANoptotic cluster was associated with some significant biological processes, we performed GSEA analysis on the GEO data and found that the PANoptotic cluster 1 was enriched in many pathways related to tumor immunity, pyroptosis, apoptosis, and necroptosis (Figures 3D,E). Since the TME plays an important role in cancer progression, we first used the “estimate” R package in the dataset to obtain the TME scores of the two clusters and then compared them. The immune score, stromal score, and ESTIMATE score values showed that there were more infiltrating immune cells and stromal cells in PANoptotic cluster 1 than in PANoptotic cluster 2 (Figure 3F and Supplementary Figure S3E). Given the difference in the level of immune cell infiltration between the two molecular patterns, we also compared the levels of 7 kinds of immune cells by EPIC between the two clusters (Figure 3G and Supplementary Figure S3F). We found that there were many kinds of immune cells, CD4 + T cells, and CD8 + T cells, with significantly different infiltration levels in the two clusters. In addition, we found that there were differences in the expression of multiple immunosuppressive checkpoint molecules between the two clusters, and their expression levels in PANoptotic cluster 1 were significantly higher than those in PANoptotic cluster 2 (Figure 3H and Supplementary Figure S3G). These findings showed that different PANoptosome patterns represented different clinical features and had different TME statuses.

### 3.4. Development and validation of a PANoptotic score model based on PANoptosome-related clusters

**Figure 4:**
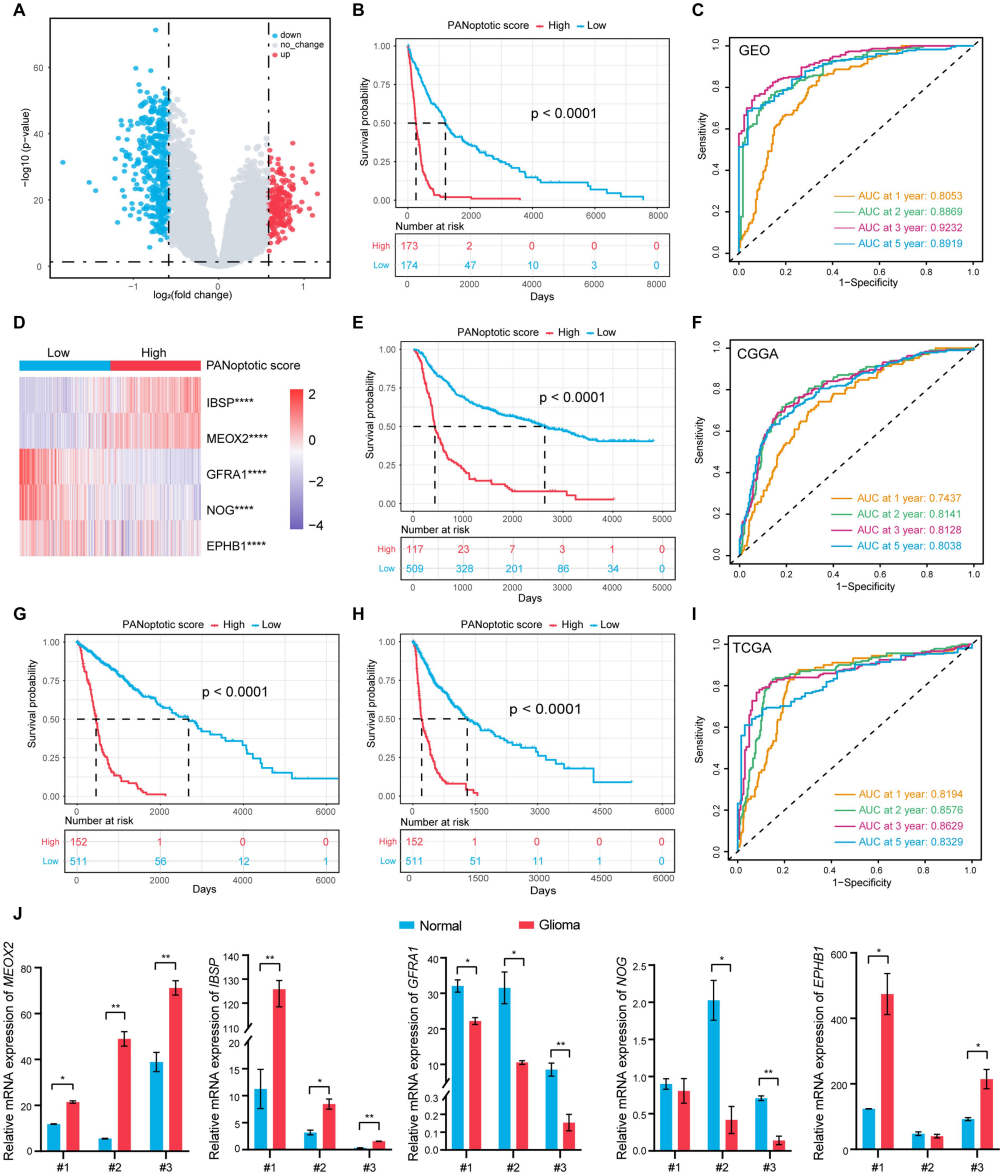
A risk score model based on PANoptotic clusters was constructed to predict the survival of glioma patients. **(A)** A summary of the differential expressed genes between two PANoptotic clusters in the combined GEO cohort (GSE16011, GSE43378, and GSE43289). **(B)** OS curves for the high and low PANoptotic score subgroups with the cut-off value 0.3658584 for 347 patients with glioma from the combined GEO cohort (GSE16011, GSE43378, and GSE43289) (Log-rank test, *p <* 0.0001). **(C)** The time-dependent receiver operating characteristic curve (ROC) of the PANoptotic score for OS. The area under the curve (AUC) was 0.8053, 0.8869,0.9232 and 0.8919 at 1-year, 2-year, 3-year, and 5-year, respectively, in the combined GEO cohort (GSE16011, GSE43378, and GSE43289). **(D)** Heatmap of the 5 genes included in the PANoptotic score model of high and low score groups in the combined GEO cohort (GSE16011, GSE43378, and GSE43289) (Student’s *t*-test, ^****^p < 0.0001). **(E)** OS curves for the different PANoptotic score subgroups with the cut-off value 0.3658584 for 626 patients with glioma from CGGA cohorts (CGGA-693 and CGGA-325) (Log-rank test, *p <* 0.0001). **(F)** The time-dependent ROC of the PANoptotic score for OS in CGGA (CGGA-693 and CGGA-325). **(G,H)** OS and PFS curves for the different PANoptotic score subgroups with the cut-off value 0,3,658,584 among 663 glioma samples from TCGA cohorts (TCGA-LGG and TCGA-GBM) (Log-rank test, *p <* 0.0001). **(I)** The time-dependent ROC of the PANoptotic score for OS in TCGA cohorts (TCGA-LGG and TCGA-GBM). **(J)** The mRNA levels of the 5 genes included in PANoptotic score in 3 pairs of glioma and the adjacent relatively normal tissues were measured by real-time PCR (Student’s *t*-test, ^*^p < 0.05; ^**^p < 0.01).

To further explore the association of PANoptosis and the prognosis of glioma patients, selecting the GEO dataset as the training cohort, we obtained 450 DEGs with an absolute value of log_2_ (FC) > log_2_ (1.5) and adjusted *p* < 0.05 related to the two PANoptotic clusters (Figure 4A). After univariate Cox regression analysis (*p* < 0.05), LASSO regression analysis, and multivariate Cox analysis (*p* < 0.05), we finally identified 5 genes independently associated with the prognosis of glioma and constructed a risk score model and named it the PANoptotic score (Supplementary Figures S4A,B and Supplementary Tables S4–6).

**Figure 5:**
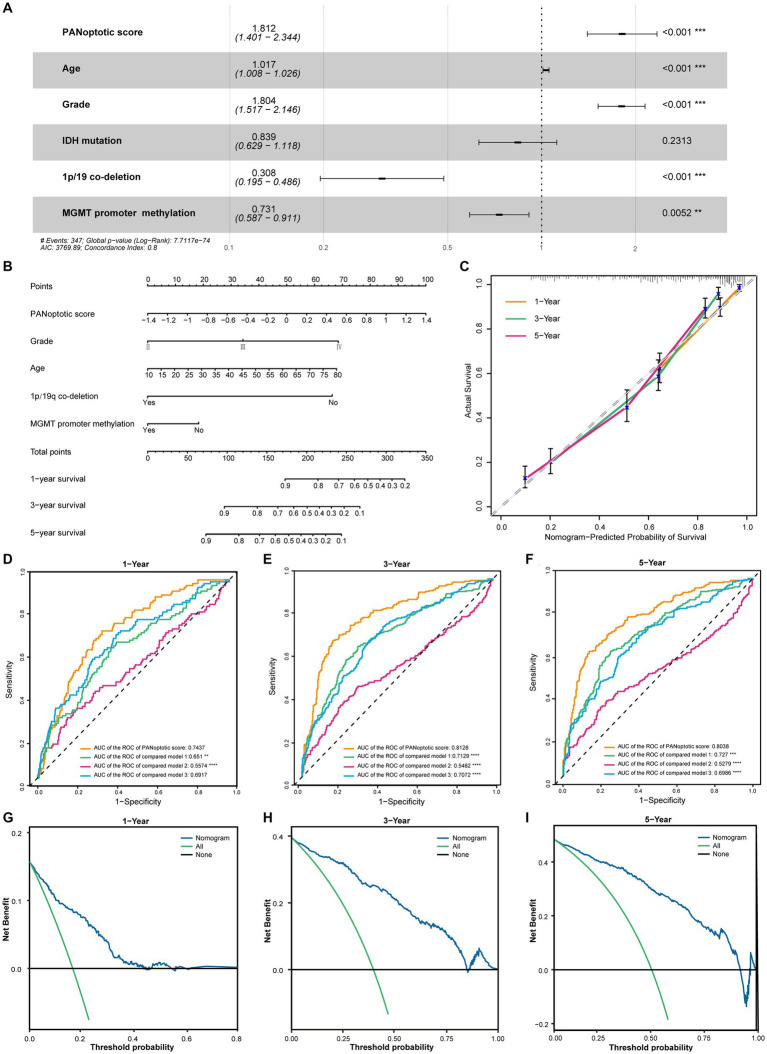
The potential clinical application of the PANoptotic score model and the construction of nomogram. **(A)** Multivariate Cox regression analysis of PANoptotic score with age, WHO grade, *IDH* mutation status, 1p19q co-deletion status, and MGMT promoter methylation status in CGGA cohorts (CGGA-693 and CGGA-325). **(B)** A nomogram based on the multivariate Cox regression analysis for clinical prognosis in CGGA cohorts (CGGA-693 and CGGA-325). **(C)** The calibration curves of the nomogram for predicting OS at 1, 3, and 5 years in CGGA cohorts (CGGA-693 and CGGA-325). The x-axis means the predicted survival probability from the nomogram, and the y-axis means the actual survival probability. **(D–F)** Comparison of time-dependent ROC of PANoptotic score and three other previously developed models to evaluate and compare their predictive accuracy at 1, 3, and 5 years in CGGA cohorts (CGGA-693 and CGGA-325). **(G–I)** Decision curve analysis (DCA) of the nomogram for 1-, 3- and 5-year risk. The x-axis shows the threshold probability, and the y-axis shows the net benefit. The black line means the assumption that no patients died at 1, 3, or 5 years. The green line means the assumption that all patients die at 1, 3, or 5 years. The blue dotted line means the prediction model of the nomogram.

Using the median value in the training dataset GEO of 0.3658584 as the cut-off value, the patients were divided into a high PANoptotic score group and a low PANoptotic score group. The heat maps visualized the differential expression of the 5 genes in the high and low PANoptotic score groups of three datasets with high statistical significance (Figure 4D and Supplementary Figures S4E,G). In the GEO dataset, by survival curves, we found that a low PANoptotic score had a better prognosis (Figure 4B). To prove that the PANoptotic score had a universal indicative value, we verified this scoring model in the CGGA and TCGA datasets with the same cut-off values and obtained the same conclusions (Figures 4E,G). In addition, in the TCGA dataset, we used the rich clinical information to plot the progression-free survival (PFS) curve and found that a low PANoptotic score in recurrence and progression of glioma also suggested a good prognosis (Figure 4H). We further found that the PANoptotic score had good predictive efficacy for patients’ survival at 1-year survival, 2-year survival, 3-year survival, and 5-year survival (Figures 4C,F,I). In the prediction of PFS, the model had equally good predictive efficacy (Supplementary Figure S4F). In order to better understand the roles of the five model genes in the occurrence and development of glioma, we tested the mRNA expression levels of the PANoptotic score model genes in the tumor tissues and adjacent relatively normal tissues. The results indicated that *IBSP*, *MEOX2*, and *EPHB1* were inclined to express highly in glioma tissues compared with the adjacent relatively normal tissues, while the expression of *NOG* and *GFRA1* tended to decrease in glioma tissues (Figure 4J). Because of the malignancy of glioma and the difficulty of treatment, researchers constructed different models, proposing some predictive markers. We compared our model with several other prognostic models to assess the importance of the PANoptotic score for predicting the prognosis of glioma. The results showed that the prediction accuracy of our model was better than other models that have been reported (Chen et al., 2021; Liu P. et al. 2022; Zeng et al., 2022) (Figures 5D–F and Supplementary Figures S5A–F).

### 3.5. There were significant differences in clinical characteristics between high and low PANoptotic score groups

In the CGGA dataset, we included PANoptotic score and clinical characteristics, such as age, WHO grade, *IDH* mutation, 1p/19q co-deletion, and MGMT promoter methylation in multivariate Cox regression analysis and found that PANoptotic score was an independent factor affecting the prognosis of glioma patients (Figure 5A). To explore the PANoptotic score in the clinical application, on this basis, we included the above clinical characteristics that independently influenced prognosis and combined with the ability of the PANoptotic score to predict the 1, 3, and 5-year survival rate of glioma patients, constructing a nomograph (Figure 5B). We further calculated the nomogram concordance index which was 0.794. The relationship between predicting OS at 1, 3, and 5 years and actual survival probability also indicated that the nomogram had a satisfactory predictive performance (Figure 5C). The decision curve suggested that the threshold probability, respectively, was 8–43%, 3–85%, and 5–92%, at 1, 3, and 5 years and the nomogram could predict survival precisely within this range (Figures 5G–I).

**Figure 6:**
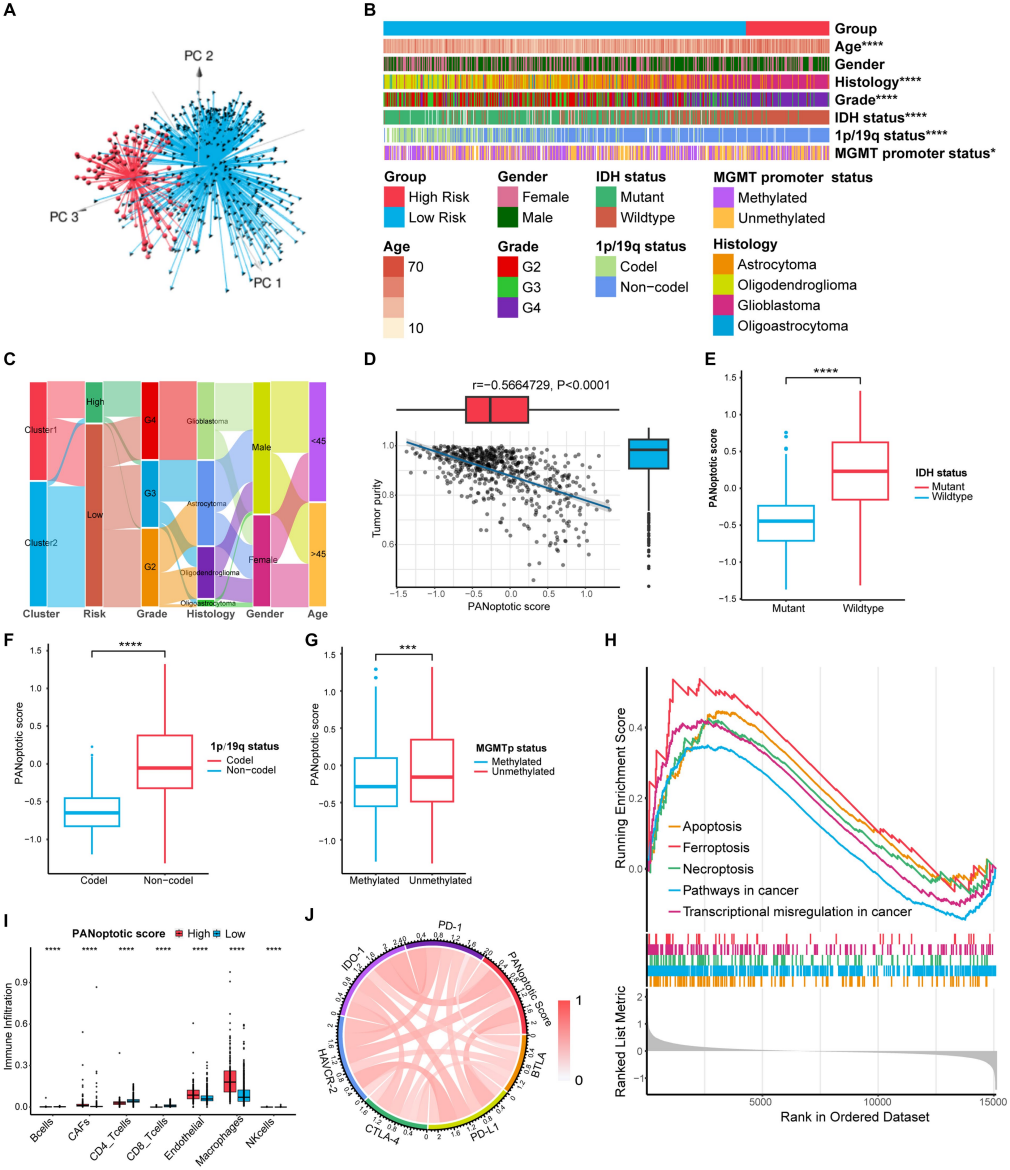
Comparison of differences in clinical features and functional enrichment between the two PANoptotic score groups. **(A)** Principal component analysis (PCA) to distinguish the high score group (*n* = 117) from the low score group (*n* = 509) in CGGA cohorts (CGGA-693 and CGGA-325). **(B)** Comparison of the distributions of clinical features between the high score group (*n* = 117) from low score group (*n* = 509) in CGGA cohorts (CGGA-693 and CGGA-325) (chi-square test, ^*^p < 0.05; ^****^p < 0.0001). **(C)** Alluvial diagram showing the changes of PANoptotic clusters, PANoptotic score, WHO grade, histology, gender, and age in CGGA cohorts (CGGA-693 and CGGA-325). **(D)** Correlation analysis of tumor purity and PANoptotic scores in CGGA cohorts (CGGA-693 and CGGA-325) (Pearson correlation coefficient). **(E–G)** Comparison of the risk scores of patients with different *IDH* mutation, 1p19q co-deletion, and MGMT promoter methylation status in CGGA cohorts (CGGA-693 and CGGA-325) (Student’s t-test). **(H)** GSEA of PANoptotic scores in the combined GEO cohort (GSE16011, GSE43378, and GSE43289). **(I)** The abundance of TME infiltrating cells between high and low score groups analyzed by EPIC in TCGA cohorts (TCGA-LGG and TCGA-GBM) (Wilcoxon test, ^****^p < 0.0001). **(J)** Analyses of the correlation between the PANoptotic scores and immune checkpoints expression in glioma patients in CGGA cohorts (CGGA-693 and CGGA-325) (Pearson correlation coefficient).

When the patients were divided into high and low-score groups using PANoptotic score, the gene expression of the subgroups could be distinguished clearly by PCA (Figure 6A and Supplementary Figures S4C,D). We analyzed the clinical characteristics of patients of the CGGA dataset in the different PANoptotic score groups. Heat maps showed statistical differences in age, WHO grade, Histology, *IDH* mutation, 1p/19q co-deletion, and MGMT promoter methylation between the two groups. We further validated it in the TCGA dataset (Figure 6B and Supplementary Figure S6A). The alluvial diagram visualized the relationship between PANoptotic clusters, PANoptotic score groups, and patient clinical characteristics of CGGA (Figure 6C). In addition, we compared several important clinical features between high and low PANoptotic score groups in the CGGA and TCGA dataset, showing that patients with three indicators of good prognosis, 1p19q co-deletion, *IDH* mutation, and MGMT promoter methylation had lower PANoptotic scores (Figures 6E–G and Supplementary Figures S6G–I). We also found that PANoptotic cluster 2, which had a clear survival advantage, and glioma patients with a lower WHO grade associated with malignancy both presented significantly lower scores. The results were consistent with our previous study (Supplementary Figures S6C–F). On the other hand, Tumor purity was negatively correlated with the PANoptotic score, providing a potential theoretical basis for the poor prognosis of the high PANoptotic score (Figure 6D and Supplementary Figure S6B).

### 3.6. The PANoptotic score could represent TME differences and provide new ideas for immune-targeted therapy

**Figure 7:**
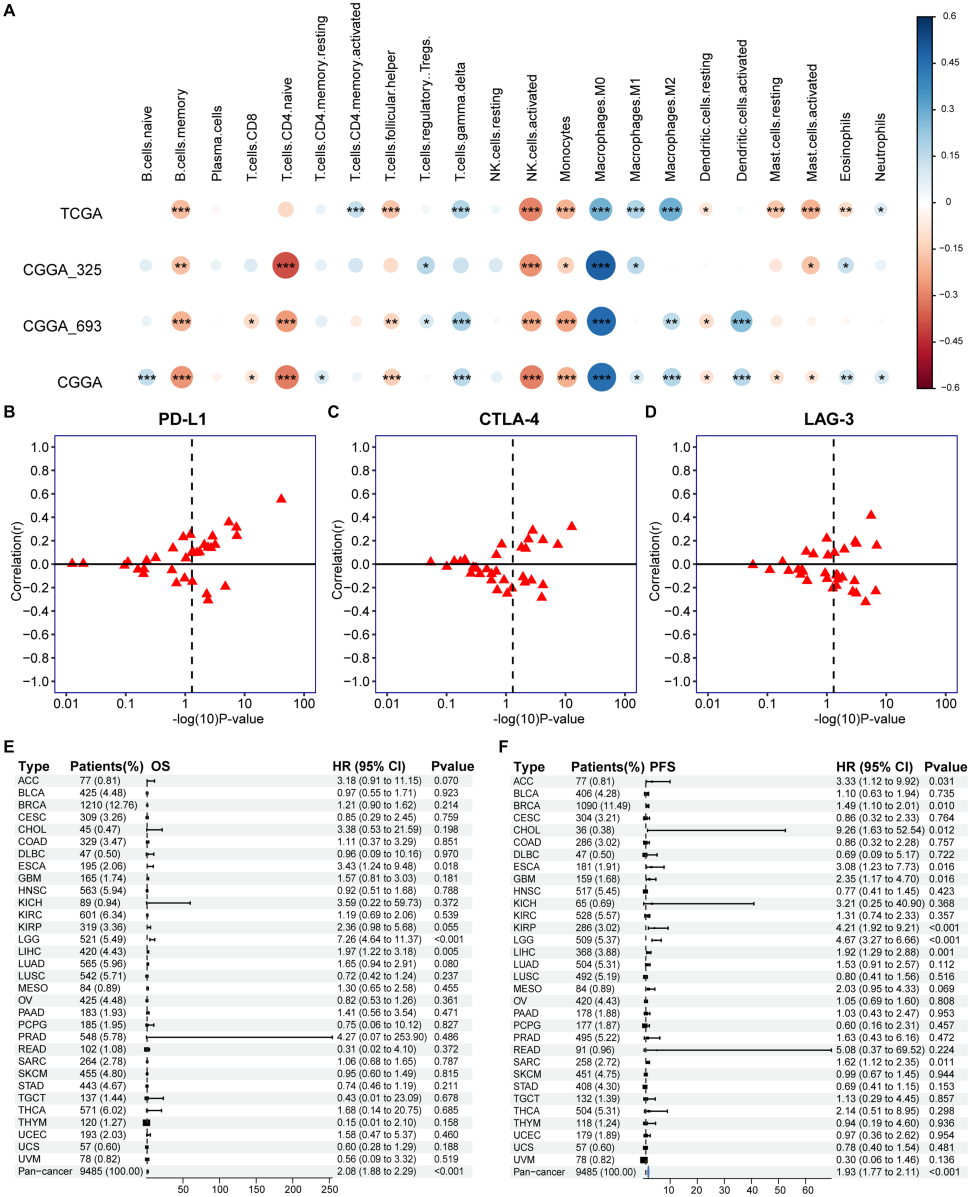
Association of PANoptotic scores with tumor microenvironment immune characteristics and prognosis at the pan-cancer level. **(A)** The correlation between TME infiltrating cells and PANoptotic scores were analyzed by CIBERSORTx in TCGA and CGGA cohorts (Pearson correlation coefficient, ^*^p < 0.05; ^**^p < 0.01; ^***^p < 0.001). Sizes of circles indicated relevant correlation coefficients. **(B–D)** Association of PANoptotic scores with immune checkpoint *PD-L1*, *CTLA-4*, and *LAG3* in diverse kinds of human tumors from TCGA database. **(E,F)** Univariate Cox regression analyses to estimate prognostic value (OS/PFS) of PANoptotic score in different cancer types from the TCGA database. The length of the horizontal line means the 95% CI for each group. The vertical dotted line means HR = 1. PFS, progression-free survival.

To research the differences in biological function between high and low PANoptotic score groups of glioma, we conducted GSEA in the GEO dataset. We found that the gene sets associated with cell death pathways and transcriptional misregulation enriched in the high PANoptotic score group. Meanwhile, gene sets related to immune cell-associated signaling pathways and *PD-1* checkpoint expression enriched in the high PANoptotic score group as well (Figure 6H, Supplementary Figures S6K,L and Supplementary Table S7). We analyzed the TME cell infiltration subsequently in the TCGA dataset, finding that there was a significant difference in the abundance of TME-infiltrating cells between the high and low PANoptotic score groups. Results showed that the low PANoptotic score group was rich in CD4+ T cells and CD8+ T cells, while the high PANoptotic score group was rich in innate immune cell infiltration including endothelial and macrophages. This suggested that the prognosis of different PANoptotic score groups may be related to TME (Figure 6I). To further guide PANoptotic score-related immune-targeted therapy, we performed a correlation analysis between PANoptotic score and immune checkpoint expression. The results of the circle graph showed that the PANoptotic score was positively correlated with *PD-L1*, *BTLA*, *PD-1*, *IDO-1*, *HAVCR-2*, and *CTLA-4* (Figure 6J and Supplementary Figure S6J). It had been suggested that *PD-1* and *PD-L1* may mediate the immune escape of tumor cells, which may also be a potential mechanism for the poorer prognosis of the high PANoptotic score group. We analyzed the correlation between PANoptotic score and immune infiltrating cells. It showed that naive CD4+ T cells, activated NK cells, Monocytes, and memory B cells had a significant negative correlation with the PANoptotic score, while M0 Macrophages, M1 Macrophages, and M2 Macrophages showed a positive correlation with the score. These all suggested that the PANoptotic score may influence the response to immunotherapy by affecting the infiltration of different types of immune cells (Figure 7A). PANoptotic score provided strategies and ideas for immune-targeted therapy of glioma while predicting prognosis.

### 3.7. The role of PANoptotic score in pan-cancer

We further extended the prognostic value of PANoptotic score to the pan-cancer level. We investigated the expression of the 5 genes included in our model in 33 tumors from the TCGA database (Supplementary Figures S7A–E). They showed expression differences between tumor tissues and normal control tissues in several cancer types, such as bladder cancer, endometrioid cancer and et al. To further validate the value of our model in guiding immunotherapy in pan-cancer, we analyzed the correlation between the PANoptotic score and the expression of several immune checkpoints in 33 tumors derived from the TCGA cohorts. We found that a high PANoptotic score is correlated with high expression of *PD-L1*, suggesting that poor prognosis of patients with high PANoptotic score may be related to tumor immune escape mediated by *PD-L1*, however, it is also suggested that patients with a high PANoptotic score may have a better response to the immunotherapy targeting *PD-L1*. The correlations between the PANoptotic score and the expression of some other immune checkpoints were also observed (Figures 7B–D and Supplementary Figures S7F–J). In addition, to validate the predictive value of PANoptotic score for OS and PFS in pan-cancer, univariate Cox regression analysis was performed on 9,485 patients of 32 tumors from the TCGA cohorts (Figures 7E,F). In the overall cohorts, a high PANoptotic score was shown to be an unfavorable prognostic factor, and the same result was seen in several independent tumor cohorts, some of which were high immunogenicity tumors, such as breast cancer, esophageal cancer, and hepatocellular carcinoma.

## 4. Discussion

The tumor is associated with dysregulation of cell death and inflammatory response (Wang et al., 2022). Genes related to PANoptosis have been mentioned by researchers in other tumors. CASPASE family members are related to the progression of tumors (Gong et al., 2022). Among them, CASPASE-8 (CASP8), as a critical protein of multiple cell death pathways, has a dual role in tumor formation and progression. It both improves the tumor microenvironment and enhances tumor autoimmunity. Meanwhile, it is involved in tumor growth and invasion, angiogenesis, and metastasis, leading to poor clinical outcomes (Jiang et al., 2021; Gong et al., 2022). In breast cancer, relevant drug treatments (e. g. DHA) can increase the apoptosis rate of breast tumor cells by increasing the expression of proteins such as AIM2 (Li et al., 2021). Methylation-mediated *PYCARD* silence helps tumor cells to escape apoptosis in breast and colorectal cancers (Sharma and Jha, 2015). In non-small cell lung cancer, the release of FADD may be involved in the metastasis of tumor cells (Cimino et al., 2012). *ZBP1* and *RIPK3* also play important roles in tumor development (Gong et al., 2022). In this study, based on PANoptosis, which is characterized by the crosstalk between the pyroptosis, apoptosis, and necroptosis of different cell death pathways (Jiang et al., 2021; Liu et al. J. 2022), we focused on the components of the PANoptosome, a protein complex that activates this cell death pathway and included 17 genes including *AIM2*, *CASP8*, *NLRP3*, *NLRP1*, *ZBP1*, etc. We found widespread alterations in the expression of the above genes in glioma patients obtained from the database and clustered patients according to 17 genes’ differential expression. In addition to differential gene expression analysis, we also analyzed copy number variations and gene mutations. Among 871 samples, 45 cases had mutations in related genes with a frequency of 5.17%. The highest mutation frequency was found in *NLRP3*, followed by *NLRP1*. CNV frequency analysis showed that CNVs were prevalent in 17 genes, with *NLPR9*, *NLPR6*, and *RIPK3* concentrating on copy number deletions, and *ZBP1* copy number amplification. Most of the remaining genes were amplified to a degree similar to the deletion.

Due to the highly infiltrative nature of glioma, patients with glioma are highly susceptible to developing resistance to currently explored treatments. Tumor recurrence and progression of malignancy are common in glioma (Lapointe et al., 2018; Wan et al., 2021). Therefore, the aggregation of genotypically diverse glioma cells into a limited epigenetically encoded transcriptional tumor phenotype may provide new therapeutic strategies and opportunities for glioma treatment (Nicholson and Fine, 2021). It is also an important direction for glioma research. At present, researchers have explored immune infiltration, methylation of genetic information, enzyme metabolism, and cell death including pyroptosis and ferroptosis, and proposed several predictive indicators that affect the prognosis of glioma and the effect of immunotherapy. While a recent paper linked PANoptosis to low-grade gliomas (LGGs) and clustered LGGs by differences in PANoptosis-associated gene expression, our study extended the relationship between PANoptosis and glioma further by breaking the limitations of grade. We analyzed the CNVs and gene mutations while studying glioma gene expression, increasing the reference value of the study. In this study, we also linked the PANoptotic score with the three molecular manifestations of 1p19q co-deletion, *IDH* mutation, and MGMT promoter methylation that are significantly related to prognosis (Weller et al., 2015). Based on enhancing the credibility of the model, the study provided new ideas for follow-up development and treatment.

According to the classification of glioma associated with PANoptosome-related genes, we constructed a risk score model containing 5 genes and divided the samples into a high-score group and a low-score group based on the PANoptotic score. In order to ensure the feasibility and theoretical basis of the model, we also conducted a preliminary investigation into the mechanism of action of these 5 genes in cell death and tumor development. *GFRA1* specifically recognizes GDNF and regulates the proliferation and differentiation of neuronal cells. Recent findings show that *GFRA1* also plays a role in cancer cell progression and metastasis (Kim and Kim, 2018). It has been reported that some types of cancers, such as breast, prostate, and lung cancers, have upregulated *IBSP* expression (Fedarko et al., 2001). *MEOX2* is a candidate oncogene that may be co-opted during tumor initiation, which cooperates with the loss of tumor suppressors *PTEN* and *p53* to promote tumor growth (Schönrock et al., 2022). In addition, the transcription factor *MEOX2* is closely associated with overall survival in glioma (Tachon et al., 2019). *EPHB1* may also be involved in the pathway of cell death. It has been shown that activation of the ephrinB1/EphB1 positive signaling pathway induces TNF-α production and may play an important role in the apoptosis of retinal ganglion cells in glaucoma (Liu et al., 2018). In tumors, EphB1/ephrins signaling has been shown to have a dual role, being involved in inhibiting tumor migration and invasion and promoting tumor progression. In particular, in GBM, patients with higher levels of *EPHB1* expression have a longer survival rate, which may be related to the enhanced positive EphB1 signaling reducing the migration and invasion of glioma (Wei et al., 2017). The accumulation of mature osteoclasts and normal bone development require the *NOG* gene. *NOG* has also been shown to be involved in bone metastasis and bone colonization of tumor cells in breast cancer (Tarragona et al., 2012; Horbinski, 2013).

In previous studies, transcriptomics-based prognostic models for glioma have also been proposed. For example, Chen et al. constructed a prognostic model of glioblastoma based on the function of STEAP (Six-transmembrane epithelial antigen of the prostate) family, STEAP2-and STEAP3-based prognostic risk score (Chen et al., 2021); Zeng et al. researched a prognostic model of glioma based on pyroptosis named pyroptosis-related risk signature (PRRS) (Zeng et al., 2022); Liu P. et al. (2022) proposed a prognostic model of glioma based on m6A regulators model called risk score, etc. The predictive performance of our constructed model was better than each of the above models by comparison of area under the ROC curve (AUC). This study showed significant survival differences between the high and low PANoptotic score groups in both the training and validation datasets, with the high PANoptotic score group having a poorer prognosis. We also compared the differences in several important clinical characteristics between high and low-score groups. The results showed that a low PANoptotic score was associated with high tumor purity, while samples with 1p19q co-deletion, *IDH* mutation, and MGMT promoter methylation have lower PANoptotic scores compared to wild-type samples. High tumor purity, 1p19q co-deletion, *IDH* mutation, and MGMT promoter methylation are all good prognostic factors for glioma. A high PANoptotic score was shown to be a poor prognostic factor. Using the information on 33 tumors from the TCGA database, we further analyzed them at the pan-cancer level and found that a high PANoptotic score also had a bad prognosis in breast cancer, esophageal cancer, hepatocellular carcinoma, and other tumors. Cell death plays an important role in tumor development. Our model linked PANoptosis, an important pathway of cell death, to the prognosis of glioma patients, providing new ideas for treating and managing glioma.

It has been shown that PANoptosis has potent immunogenicity (Liu J. et al. 2022). It can enhance the anti-tumor immunity in TME by activating the immune system to kill tumor cells and overcome the problem of drug resistance during treatment if other PCD pathways are suppressed or mutated (Jiang et al., 2021; Liu J. et al. 2022). It has been demonstrated that TNF-α and IFN-γ can combine to enhance anti-tumor immunity by activating PANoptosis (Malireddi et al., 2021). However, research on anti-tumor immunity of PANoptosis is still in the exploratory stage. In this study, we compared the differences in immune infiltration between the high-score group and low-score group of the constructed model, explored the correlation between PANoptotic score and the expression of several major immune checkpoints, and extended the model to the pan-cancer level by analyzing the correlation between PANoptic score and immune checkpoints *CD274*, *CTLA-4* and *LAG3* in some other tumors. The current study could provide preliminary evidence that the PANoptotic score of our model had significant value in assessing patients’ responsiveness to immunotherapy and predicting outcomes, further providing guidance for immune-targeted therapy for glioma and other tumors. Although the model we constructed analyzed the prognosis of glioma and the direction of immune-targeted therapy from multiple aspects and levels, the deep molecular mechanisms need to be further explored.

In this study, we found significant changes in PANoptosome-related genes at both the gene molecular level and transcriptional expression level. Based on this, we obtained two clustering patterns using unsupervised clustering, proposed a molecular subtype of glioma associated with PANoptosis, and explored to find statistically significant differences in clinical features, immune infiltration, and biological behaviors of patients between the two patterns. The results suggested that PANoptosome-related genes may reveal the internal features of glioma and had potential implications for the molecular genetic characteristics and tumor heterogeneity of glioma. We constructed a prognostic assessment model based on the differential genes of the two PANoptotic clusters and validated the prognostic efficacy of the PANoptotic scores on glioma in multiple datasets. We combined PANoptotic scores with several clinical features that independently affected glioma prognosis to construct the nomograph and further investigated the possible value of the model in clinical applications. Finally, we extended the PANoptotic scores to the pan-cancer level, providing a deeper direction for exploring the PANoptosis-related mechanisms of a variety of tumors and providing new ideas for the generation of breakthrough therapies such as immune-targeted therapy.

## Data availability statement

The original contributions presented in the study are included in the article/Supplementary material, further inquiries can be directed to the corresponding author. The raw data can be found at: https://www.jianguoyun.com/p/DTAP4gEQ5MPHCxjXuYAFIAA.

## Ethics statement

The studies involving human participants were reviewed and approved by The Ethical Committee of Medical Integration and Practice Center, Shandong University. The patients/participants provided their written informed consent to participate in this study.

## Author contributions

XL designed the study. JS, ZX, QF, and YS collected and analyzed the data and performed the expression verification of clinical samples. XL, JS, ZX, and QF wrote the manuscript. XL supervised the study. All authors contributed to the article and approved the submitted version.

## Funding

This study was supported by the Medical and health technology development program of Shandong province (2016ws0126) and the Clinical Research Fund of Shandong Medical Association-Qilu Special Project (YXH2022ZX02195).

## Conflict of interest

The authors declare that the research was conducted in the absence of any commercial or financial relationships that could be construed as a potential conflict of interest.

## Publisher’s note

All claims expressed in this article are solely those of the authors and do not necessarily represent those of their affiliated organizations, or those of the publisher, the editors and the reviewers. Any product that may be evaluated in this article, or claim that may be made by its manufacturer, is not guaranteed or endorsed by the publisher.

## Footnotes

^1^ https://cibersortx.stanford.edu/

^2^ http://epic.gfellerlab.org/
