# Supplementary figures and images for: The PANoptosis-related signature indicates the prognosis and tumor immune infiltration features of gliomas
Source: Front Mol Neurosci. 2023 Jul 12;16:1198713. doi: 10.3389/fnmol.2023.1198713 (PMC10369193; doi:10.3389/fnmol.2023.1198713)

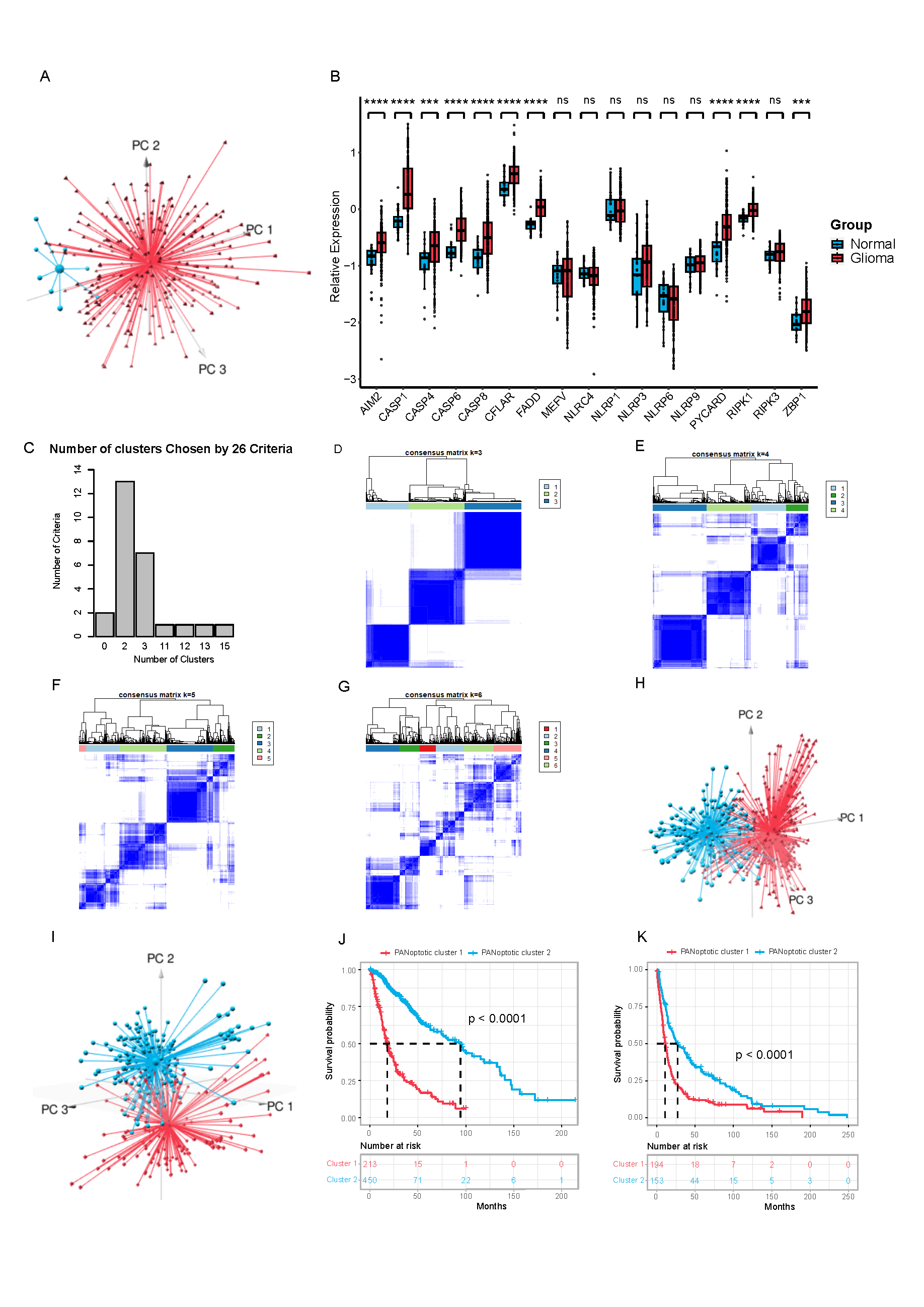

Supplement: Supplementary file 2 [file Image_1.TIF]

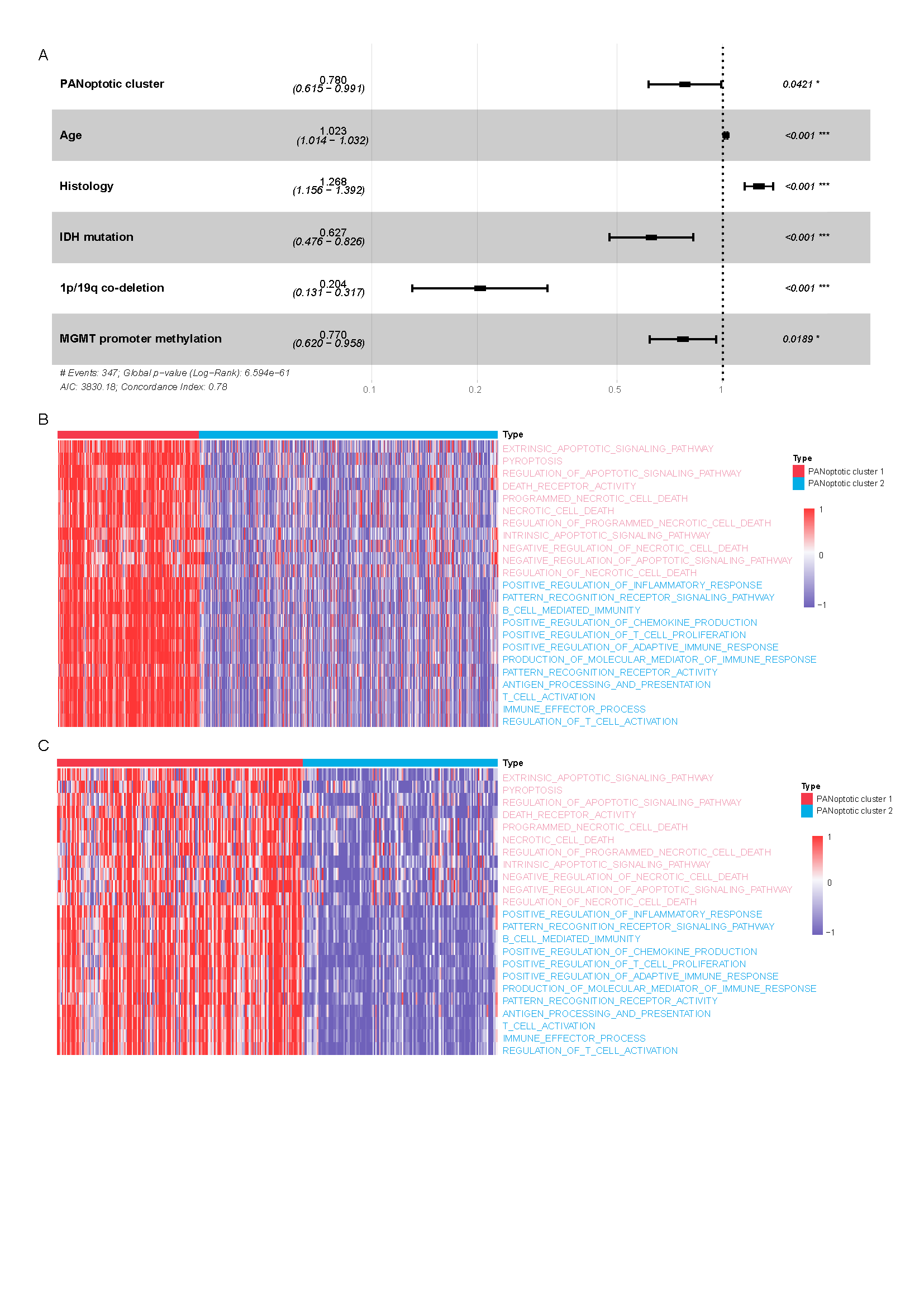

Supplement: Supplementary file 3 [file Image_2.TIF]

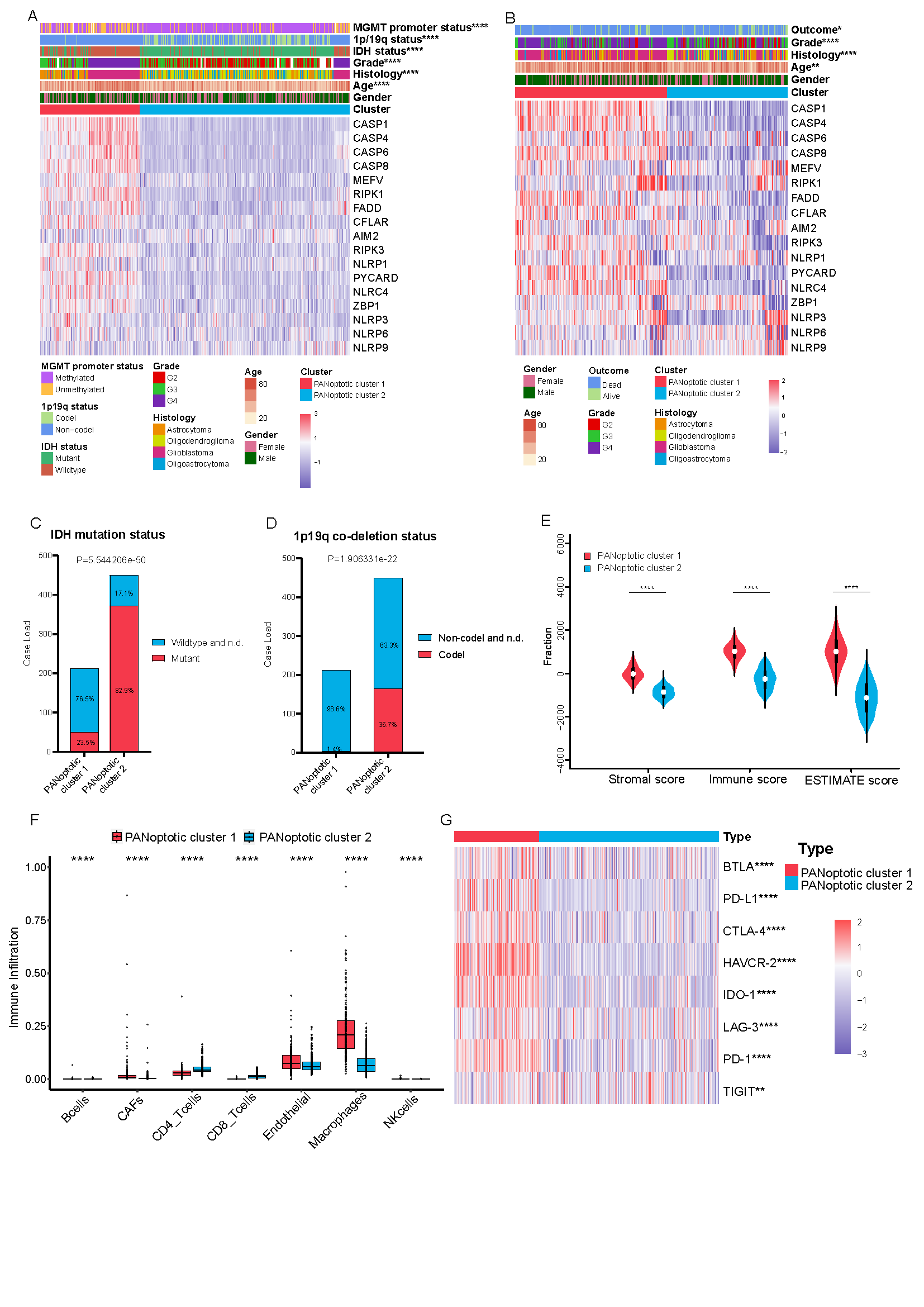

Supplement: Supplementary file 4 [file Image_3.TIF]

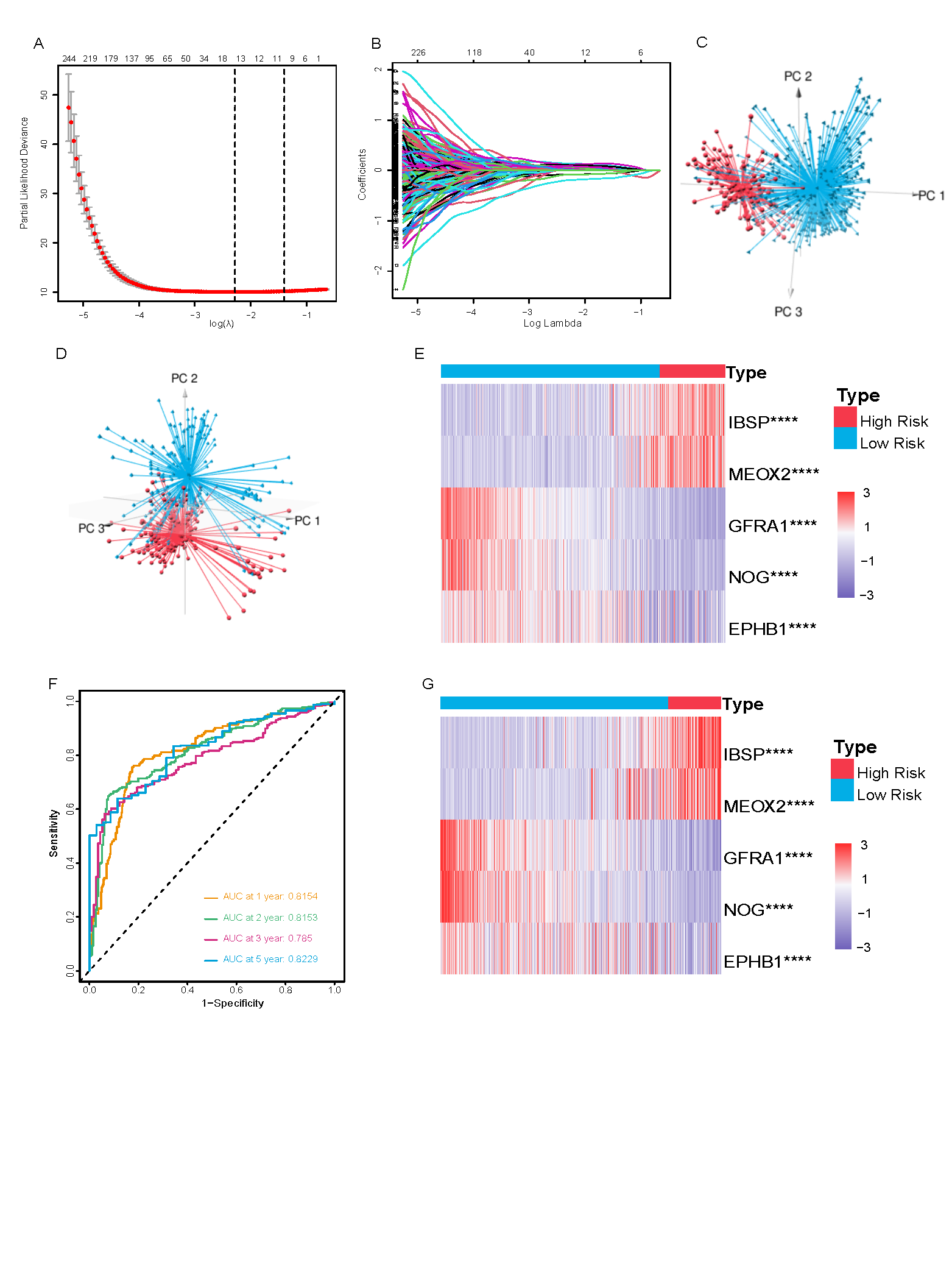

Supplement: Supplementary file 5 [file Image_4.TIF]

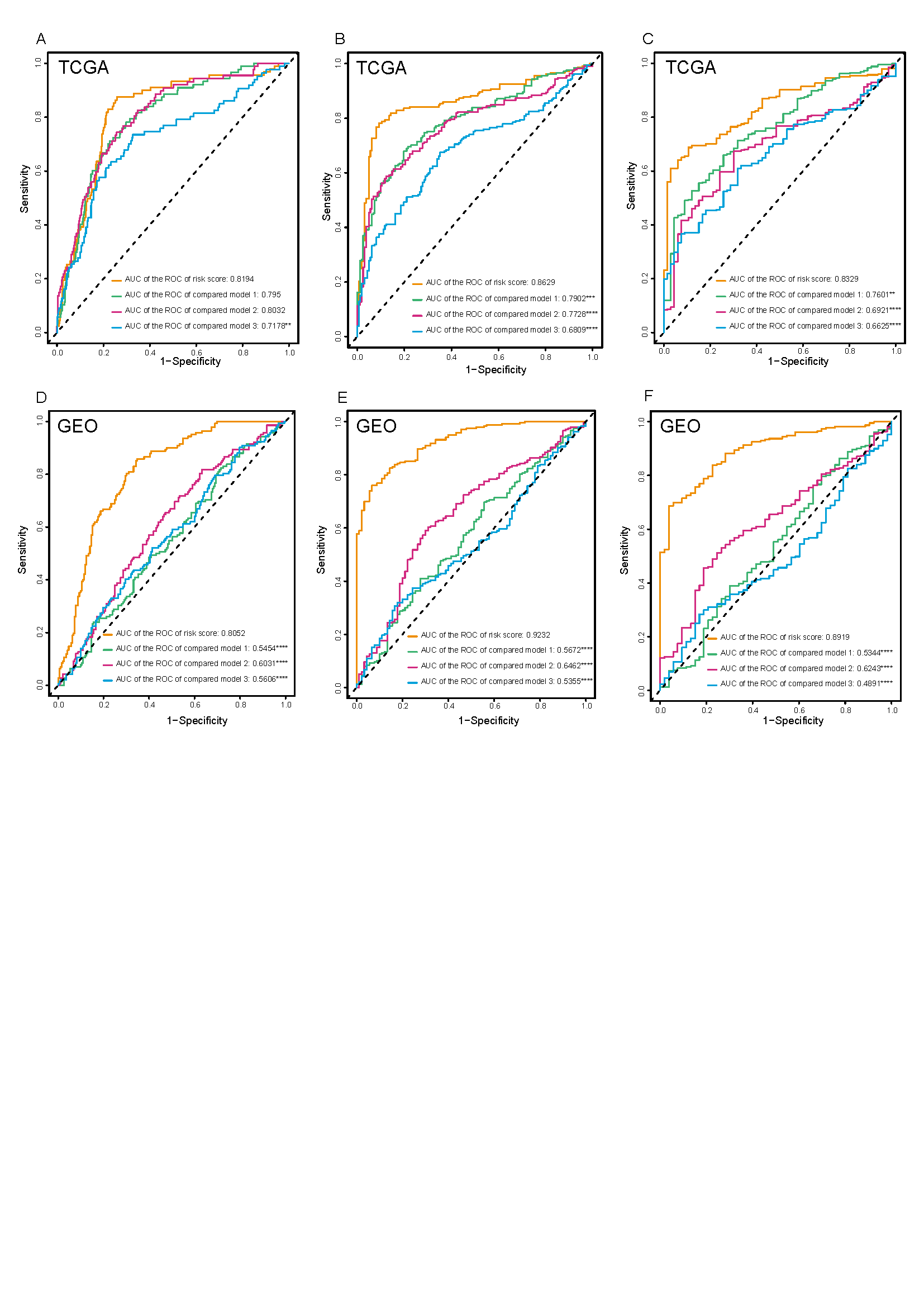

Supplement: Supplementary file 6 [file Image_5.TIF]

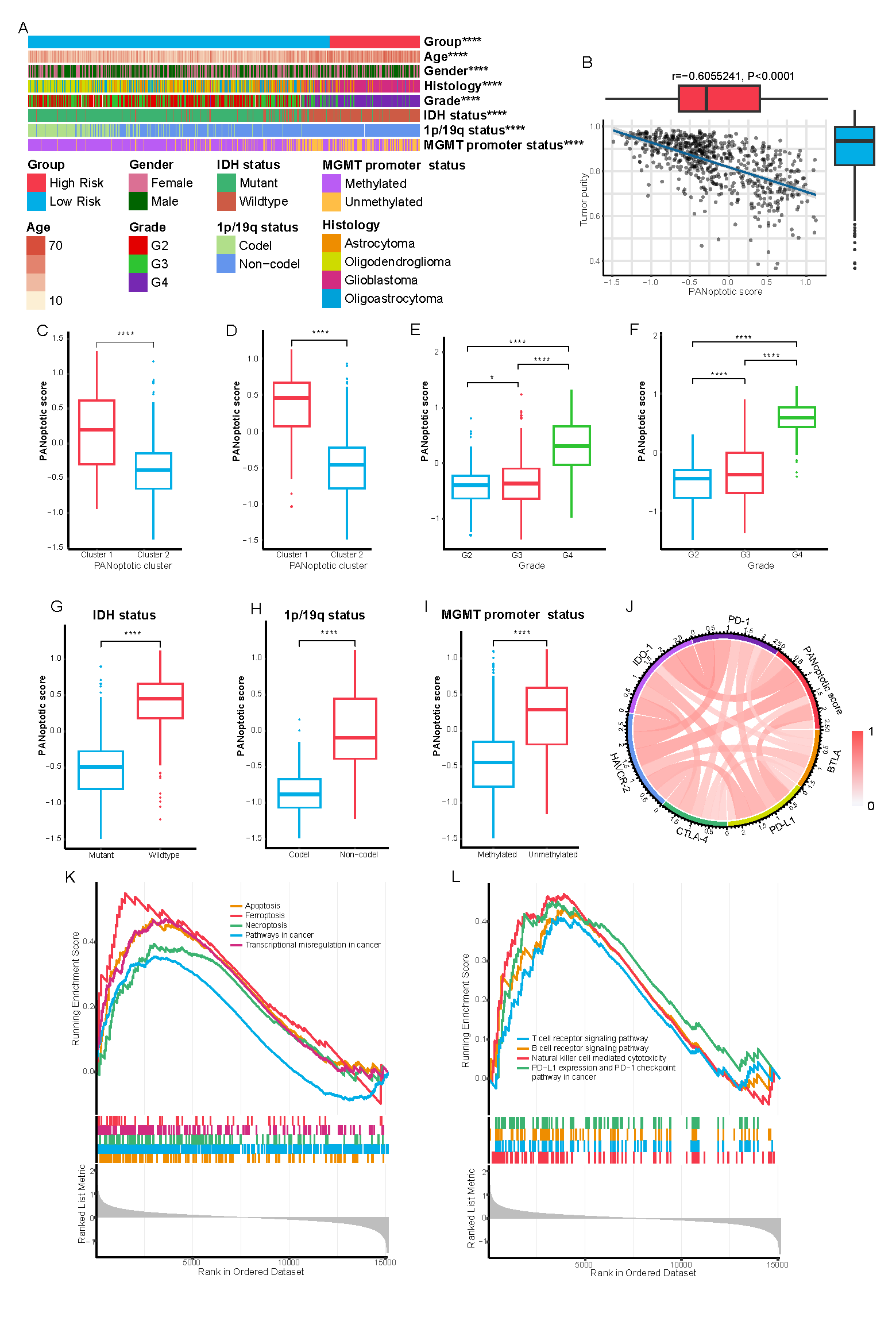

Supplement: Supplementary file 7 [file Image_6.TIF]

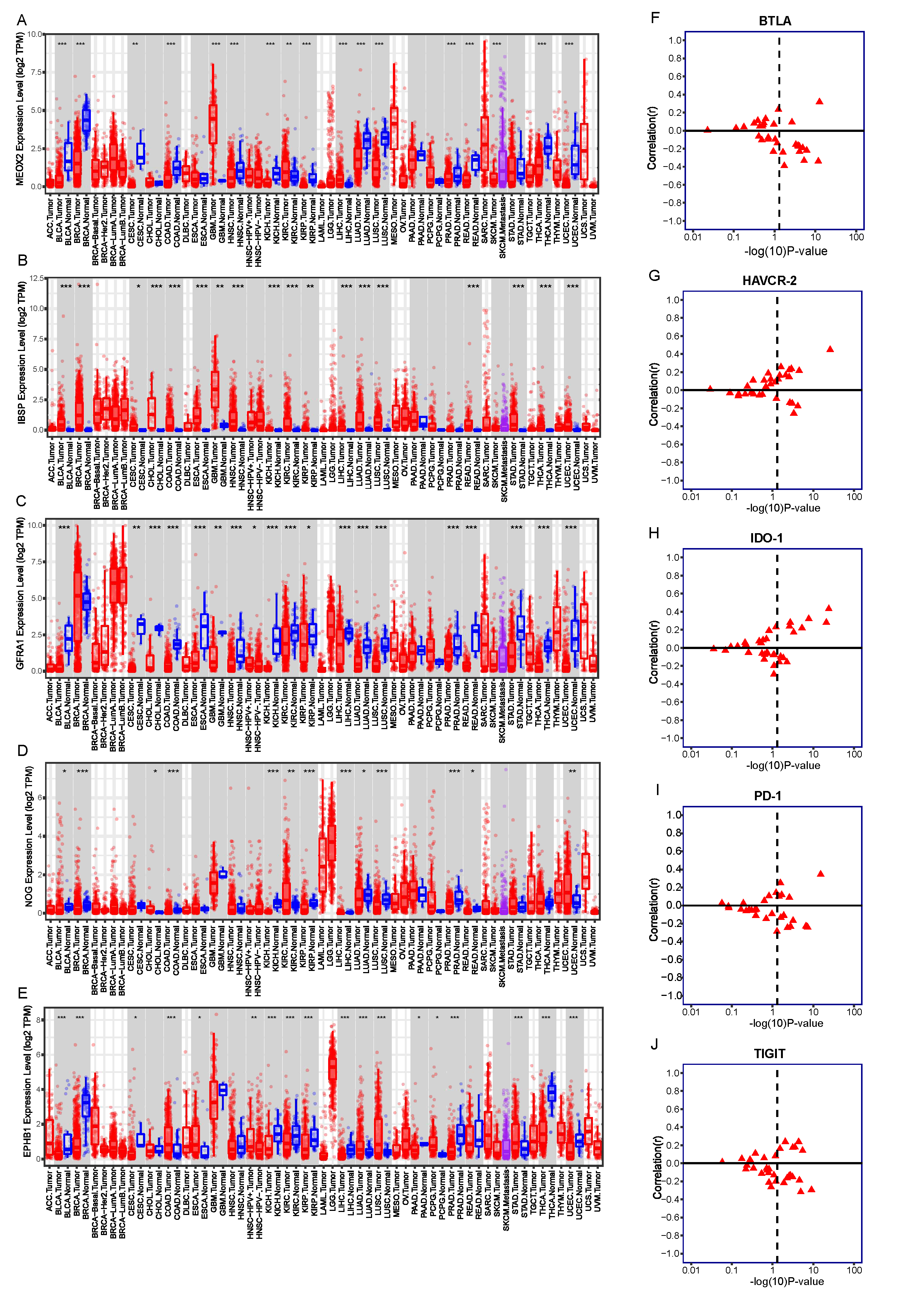

Supplement: Supplementary file 8 [file Image_7.TIF]
